# 非变性质谱和紫外激光解离揭示锌离子对α-突触核蛋白的构象选择性

**DOI:** 10.3724/SP.J.1123.2025.04020

**Published:** 2026-05-08

**Authors:** Huiwen QIN, Yue XUAN, Heng ZHAO, Zheyi LIU, Fu DING, Fangjun WANG

**Affiliations:** 1.沈阳化工大学化学工程学院，辽宁 沈阳 110142; 1. College of Chemical Engineering，Shenyang University of Chemical Technology，Shenyang 110142，China; 2.化学反应动力学全国重点实验室，中国科学院大连化学物理研究所，辽宁 大连 116023; 2. State Key Laboratory of Molecular Reaction Dynamics，Dalian Institute of Chemical Physics，Chinese Academy of Sciences，Dalian 116023，China

**Keywords:** α-突触核蛋白, 锌离子, 紫外激光解离, α-synuclein （α-Syn）, zinc ion （Zn^2+^）, ultraviolet photodissociation （UVPD）

## Abstract

锌离子（Zn^2+^）与α-突触核蛋白（α-Syn）的构象特异性结合机制对揭示帕金森病发病机理具有重要意义。本研究通过非变性质谱（nMS）联用紫外激光解离（UVPD）技术，揭示了α-Syn的3种构象异质性，并鉴定出具有显著Zn^2+^结合差异的低价态、中间价态及高价态构象。实验数据显示，低价态构象表现出最强的Zn^2+^结合能力，其结合位点数超过2个且结合强度显著高于仅结合1个Zn^2+^的中间价态构象和结合2个Zn^2+^的高价态构象。进一步通过解离效率差异（ΔFYs）的定量比较发现，Zn^2+^结合诱导不同构象的α-Syn发生不同的结构变化，其中低价态构象的ΔFYs变化最为显著。对UVPD产生的Zn^2+^结合蛋白碎片（holo碎片）进行分布模式分析表明，3种构象中的holo碎片分布存在显著差异。结合一级谱分布特征、ΔFYs的变化特征和holo碎片分布特征，可以推测出低价态构象主要依赖C端（Cterm）的静电相互作用形成Zn^2+^-蛋白复合物，中间价态构象以配位结合为主导，而高价态构象则表现出两种作用模式的协同效应。该研究从多构象动态平衡视角阐明了α-Syn-Zn^2+^相互作用的分子机制，为针对蛋白质构象特异性设计帕金森病调控疗法提供了理论依据。

## Abstract (en)

The conformation-specific binding mechanism between zinc ions （Zn^2+^） and α-synuclein （α-Syn） plays a pivotal role in elucidating the molecular underpinnings of Parkinson’s disease. α-Syn is an intrinsically disordered protein （IDP）， known for its structural plasticity and dynamic conformational landscape， which are intimately linked to its aggregation propensity and pathogenic potential. In this study， we employed native mass spectrometry （nMS） coupled with 193 nm ultraviolet photodissociation （UVPD） to investigate the α-Syn-Zn^2+^ interaction across different conformational states. This integrated approach enables both preservation of non-covalent interactions and detailed structural interrogation， offering unparalleled insights into IDP behavior upon metal binding. We identified three distinct charge-state-dependent conformational populations of α-Syn （low-charge， intermediate-charge， and high-charge conformations）， each exhibiting markedly different Zn^2+ ^binding capacities and mechanisms. Experimental data revealed that the low-charge conformations exhibited the highest Zn^2+ ^binding affinity and capacity， accommodating up to three Zn^2+^ ions. In contrast， the intermediate-charge conformations bound predominantly to one Zn^2+^ ion. The high-charge conformations， despite their extended structures， retained the ability to bind up to two Zn^2+^ ions， but with lower affinity. Quantitative analysis of UVPD-derived fragmentation yield changes （ΔFYs） provided residue-level insights into structural perturbations upon Zn^2+^ coordination. The most pronounced ΔFYs were observed in the low-charge conformers， indicating substantial Zn^2+^-induced structural stabilization or reorganization， particularly in the C-terminal （Cterm）. Distribution patterns of Zn^2+^-bound protein fragments （holo fragments） generated by UVPD further supported distinct fragmentation patterns for each conformational state， reflecting differential Zn^2+^ distribution and protection across the α-Syn sequence. Integrating ΔFYs analysis with holo fragment mapping， we propose three distinct binding mechanisms： （i） low-charge states stabilize Zn^2+^ binding primarily through electrostatic interactions involving acidic residues in the Cterm； （ii） intermediate-charge states form coordination bonds likely involving histidine or side-chain donors； and （iii） high-charge states exhibit a hybrid mechanism combining electrostatic and coordination elements， though with reduced spatial proximity and structural integrity. Overall， this work highlights the conformation-dependent nature of metal ion interactions in IDPs and underscores the potential of nMS-UVPD as a powerful tool for probing dynamic structural ensembles. These findings provide critical mechanistic insights that could inform the design of conformation-selective therapeutic agents aimed at modulating metal-induced α-Syn aggregation in Parkinson’s disease.

α-突触核蛋白（α-Syn）作为一类典型的固有无序蛋白（IDPs），在神经元生理功能维持及帕金森病（PD）等神经退行性疾病的发病机制中具有重要作用^［1］^。其生理功能主要包括调控突触囊泡的循环过程、介导神经递质的释放以及参与细胞信号转导^［2］^。然而α-Syn异常构象转换并形成不可溶性纤维沉积时，可导致神经元损伤并促进疾病进展^［3］^。α-Syn由140个氨基酸（AA）组成^［4］^，其各结构域具有明显的功能分化：N端（Nterm）（AA1~60）呈现两亲性特征，可与脂类结合形成α螺旋结构^［5］^；疏水性非淀粉样核心区段（NAC）（AA61~95）是蛋白低聚化及纤维化形成的关键区域^［6，7］^；酸性C端（Cterm）（AA96~140）则具有金属离子结合特性^［8，9］^。大量研究证实，α-Syn与金属离子的相互作用通过调控蛋白构象稳定性影响其聚集路径，这一分子机制已成为神经化学及结构生物学领域的重要研究方向^［8，10］^。

锌离子（Zn^2+^）是人体必需的微量元素，在细胞代谢、增殖及凋亡等基础生命活动中发挥关键的调控作用^［10］^。在神经系统中，Zn^2+^不仅参与突触信号传递和神经可塑性调节，更显示出独特的神经保护功能^［10］^。研究表明，Zn^2+^能够通过与α-Syn的特异性相互作用，精确调控该蛋白的结构稳定性与生理功能^［10］^。然而，当Zn^2+^与α-Syn异常结合时，会诱导蛋白错误折叠，促进病理性寡聚体及淀粉样纤维的形成，从而增强其神经毒性效应^［10］^。现有研究通过多种表征技术（包括核磁共振波谱、圆二色光谱、荧光光谱及质谱分析等），已系统阐明Zn^2+^的结合位点主要位于α-Syn的中间结构域（AA40~80）和Cterm区域（AA96~140）^［11-16］^。值得注意的是，生理浓度范围内的Zn^2+^可有效维持α-Syn的构象稳定性，但当Zn^2+^浓度超过生理阈值时，会显著促进其病理性聚集，形成具有神经毒性的纤维结构^［11-16］^。

尽管现有研究已采用多种实验手段探究Zn^2+^-α-Syn的相互作用机制，但传统结构解析技术仍存在局限性。固体核磁共振（ssNMR）和X射线衍射（XRD）由于依赖样品结晶特性，难以捕捉无固定结构的α-Syn单体及低聚体动态构象变化^［6，17］^；透射电镜（TEM）虽可观测纤维形貌，却无法解析早期聚集中间态的结构特征^［6，17］^。此外，由于α-Syn单体和低聚体具有多种构象，传统光谱技术受限于异质体系的信号叠加效应，难以实现构象的分离检测，而单分子技术虽具备单粒子分辨率，但其对蛋白精细结构的解析能力仍存在瓶颈^［18，19］^。

在此背景下，非变性质谱（nMS）因其高效的分离能力，可基于构象带电差异精准区分不同构象，进一步结合离子淌度（IM）、电子转移解离（ETD）等手段，可深入解析α-Syn的构象特征^［20，21］^。例如，nMS-IM联用分析表明，α-Syn单体存在三类典型构象亚群，nMS-ETD联用实验结果进一步表明，α-Syn的二聚体的相互作用界面可能位于Cterm^［12，21］^。此外，nMS结合电子捕获解离（ECD）/碰撞诱导解离（CID）分析显示，锰和钴离子在α-Syn上的结合位点相同，均位于Cterm区域^［22］^；nMS-ECD技术还进一步发现，小分子配体（如CLR01）可调控铜、锰等金属离子与α-Syn的结合能力^［23］^。分子动态学研究方面，氢氘交换质谱（HDX-MS）实验证实α-Syn的Nterm结构域暴露程度与其聚集动力学呈正相关性，同时该区域的构象可塑性还直接影响钙离子结合的调控能力^［4］^。紫外光解离（UVPD）技术通过高能量的紫外光子激活和解离前体离子，相较于其他解离方法，不仅能获得更高的序列覆盖度，还可完整保留配体结合后的蛋白碎片（holo碎片），为研究金属离子-蛋白复合物的结合构象提供了独特技术优势^［24，25］^。

本研究采用nMS技术揭示了α-Syn与Zn^2+^结合的构象选择性，并基于UVPD技术探究了Zn^2+^结合对不同α-Syn构象的调控作用。实验数据显示，不同α-Syn构象结合Zn^2+^的个数及强度不同。进一步分析表明，Zn^2+^结合虽显著改变各构象的结构特性，但未引起低、中、高价态构象亚群的相对丰度发生显著变化。本研究不仅有助于深入理解金属-α-Syn的相互作用机制，还为神经退行性疾病的药物开发提供了新视角。

## 1 实验部分

### 1.1 仪器、试剂与材料

AKTA高效液相色谱纯化系统和Superdex 200 Increase 10/300 GL尺寸排阻色谱柱（SEC）购自Cytiva公司（英国）；Orbitrap Fusion Lumos Tribrid三合一超高分辨质谱分析系统购自ThermoFisher公司（美国）；193 nm ArF准分子激光器购自Coherent Laser Systems Gmbh & Co.KG（德国）。

pET-22b质粒、大肠杆菌BL21（DE3）和氯化钠（NaCl）均购自Sangon公司（中国）。氨苄青霉素、琼脂、酵母提取物、胰蛋白胨和异丙基-*β*-D-硫代半乳糖苷（IPTG）均购自BBI生命公司（中国）。三羟甲基氨基甲烷（Tris）购自上海吉至公司（中国）。盐酸、乙二胺四乙酸（EDTA）、硫酸链霉素和硫酸铵购自上海阿拉丁公司（中国）。醋酸铵购自Sigma-Aldrich公司（美国）。醋酸锌购自迈瑞尔公司（中国）。P6脱盐柱购自伯乐公司（美国）。熔融石英毛细管购自Polymicro Technologies公司（美国）。透析袋（分子质量截断值为3 kDa）和0.5 mL/15 mL超滤管（分子质量截断值为3 kDa）购自Merck公司（美国）。

### 1.2 α-Syn的重组表达与分离纯化

使用pET-22b质粒和大肠杆菌BL21（DE3）重组表达α-Syn。将重组细菌接种于含100 mg/L氨苄青霉素的Luria-Bertani（LB）培养基（胰蛋白胨10 g/L、酵母提取物5 g/L、NaCl 10 g/L、琼脂15 g/L）中，37 ℃过夜培养。挑取培养基上的单菌落转入15 mL的 LB培养液管中进行小规模培养（37 ℃，8~12 h），然后转入2 L LB培养液瓶进行大规模培养。37 ℃培养8~12 h后，加入终浓度为0.5 mmol/L的IPTG诱导蛋白表达，30 ℃诱导6~8 h。细菌经3 500 g离心20 min收集，沉淀重悬于裂解缓冲液（20 mmol/L Tris pH 8.0、1 mmol/L EDTA）中并进行超声破碎。裂解液在4 ℃下以12 000 g离心25 min，上清液在85 ℃下孵育10 min，再以11 000 g离心20 min。收集上清液后，加入过饱和硫酸链霉素搅拌20 min，再离心（11 000 g，20 min）。取上清继续加入硫酸铵至饱和，搅拌20 min后，再次离心（11 000 g，20 min），收集沉淀。沉淀重悬于20 mL裂解缓冲液中，并在50 mmol/L醋酸铵溶液中透析4轮，每轮至少2 h。透析后的溶液用15 mL超滤管离心浓缩。浓缩液使用SEC分离，流动相为20 mmol/L Tris（pH 7.4）、1 mmol/L EDTA、100 mmol/L NaCl，流速为1 mL/min，在280 nm监测紫外吸收。纯化后的蛋白样品储存在流动相中，并于-20 ℃保存备用。

### 1.3 样品制备

在进行nMS-UVPD分析之前，需对α-Syn样品进行缓冲液置换。具体操作：使用P6脱盐柱或0.5 mL超滤离心管，将样品中的原始缓冲液更换为500 mmol/L的醋酸铵缓冲液。缓冲液置换完成后，进一步通过稀释或浓缩步骤将蛋白质浓度调整至20 μmol/L，以满足后续质谱分析的需求。α-Syn与Zn^2+^按物质的量之比为1∶50孵育2 h以上，使用500 mmol/L的醋酸铵作为孵育缓冲液，并将样品保存在4 ℃。

### 1.4 nMS-UVPD分析

对每种蛋白质溶液，电喷雾电压的最佳电压为1 400 V左右。所有实验均在配备193 nm 紫外激光解离技术的Orbitrap Fusion Lumos Tribrid质谱仪上进行。所有一级质谱图均采用Orbitrap质量分析器以24万分辨率采集。不同状态的蛋白质离子采用四极杆进行隔离选择，隔离窗口设置为*m/z* 2，随后采用单脉冲 193 nm 紫外激光激发（脉冲能量：1.2 mJ左右），产生的碎片离子采用Orbitrap质量分析器以50万分辨率采集。

### 1.5 质谱数据分析

针对电荷态分布（CDSs）的分析，从原始质谱数据（*.raw文件）中提取α-Syn结合Zn^2+^前后不同价态的强度及电荷信息。随后将这些数据导入OriginPro 2025软件，采用该软件提供的Peak Analyzer中的Gauss函数拟合方法进行拟合分析，通过Fit converged获得收敛的最佳拟合解。

针对解离碎片的分析，质谱采集的Raw文件采用MSConvert （版本3.0）转换为mzML格式^［26］^。随后，使用TopFDGUI（版本1.6.2）进行去卷积处理^［27］^，信噪比阈值设定为3。去卷积后的数据包含碎片离子的单同位素质量，使用计算机生成的数据库进一步校准并搜索，范围涵盖所有可能的碎片离子（a、a+1、a+2、b、c、x、x+1、y、y-1、y-2、z、z+1），相对质量误差不超过2×10^-6^。使用皮尔逊相关系数评估理论同位素分布与实验数据的相关性，相关系数>0.7的离子视为可靠离子。每个重复中含20次扫描，过滤检测到的扫描次数小于16的碎片离子。通过匹配检测到的同位素峰与计算出的同位素峰的轮廓，开发了一个扫描匹配函数。此过程仅针对具有相同电荷态的α-Syn/α-Syn-Zn^2+^数据。在去卷积过程中，手动检查重叠的单同位素峰的对应碎片离子。最后，通过中位数归一化对鉴定到的碎片离子强度进行归一化。将所有碎片离子的强度归一化计算碎片产率（FYs）。将a、a+1、a+2、x、x+1离子的相对强度相加，确定每个残基位点的UVPD解离FYs，随后绘制蛋白质序列各位点的对应FYs变化图。不同条件间FYs差异（ΔFYs）的统计显著性通过*t*检验确定，并绘制以每个位点的ΔFYs。*P*≤0.001的位点被认为具有显著性差异。holo碎片离子的识别及比例计算与其他碎片离子类似，但使用的离子类型包括a、a+1、a+2、b、c、x、x+1、y、y-1、y-2、z和z+1离子，同一位点的同端holo碎片视为相同holo碎片。所有数据分析均通过自定义R脚本完成（版本4.4.0）。本研究中使用的原始数据已公开储存在Zenodo数据存储库（https：//doi.org/10.5281/zenodo.15362532），读者可通过链接直接获取。

## 2 结果与讨论

### 2.1 **α**-Syn单体的构象异质性及其对Zn^2+^的选择性

对α-Syn一级质谱数据的拟合结果显示，CDSs可分解为3个正态分布的峰，峰值分别位于7价（7+）、10价（10+）和13价（13+）（图1a）。根据nMS结果，α-Syn的构象可被分为三类：低价态、中间价态和高价态构象亚群，这一分类与离子迁移谱（IM）分析结果一致^［12］^。在Zn^2+^结合实验中，我们采用物质的量之比为1∶50的α-Syn/Zn^2+^，经2 h孵育后进行nMS检测。质谱分析表明，所有价态均能与Zn^2+^结合，且α-Syn与Zn^2+^结合后，其CDSs同样可拟合为3个正态分布的峰（图1b），对应的低、中、高价态构象亚群的相对丰度变化较小。此外，nMS结果还表明*α*-Syn与Zn²⁺的结合特性随其价态变化而呈现出差异：在低价态构象中，*α*-Syn结合的Zn²⁺数量最多，结合强度最高；在中价态构象中，结合的Zn²⁺数量最少，结合最弱；而在高价态构象中，其结合的Zn²⁺数量及结合强度均介于低、中两种价态之间（图2）。

**图1:**
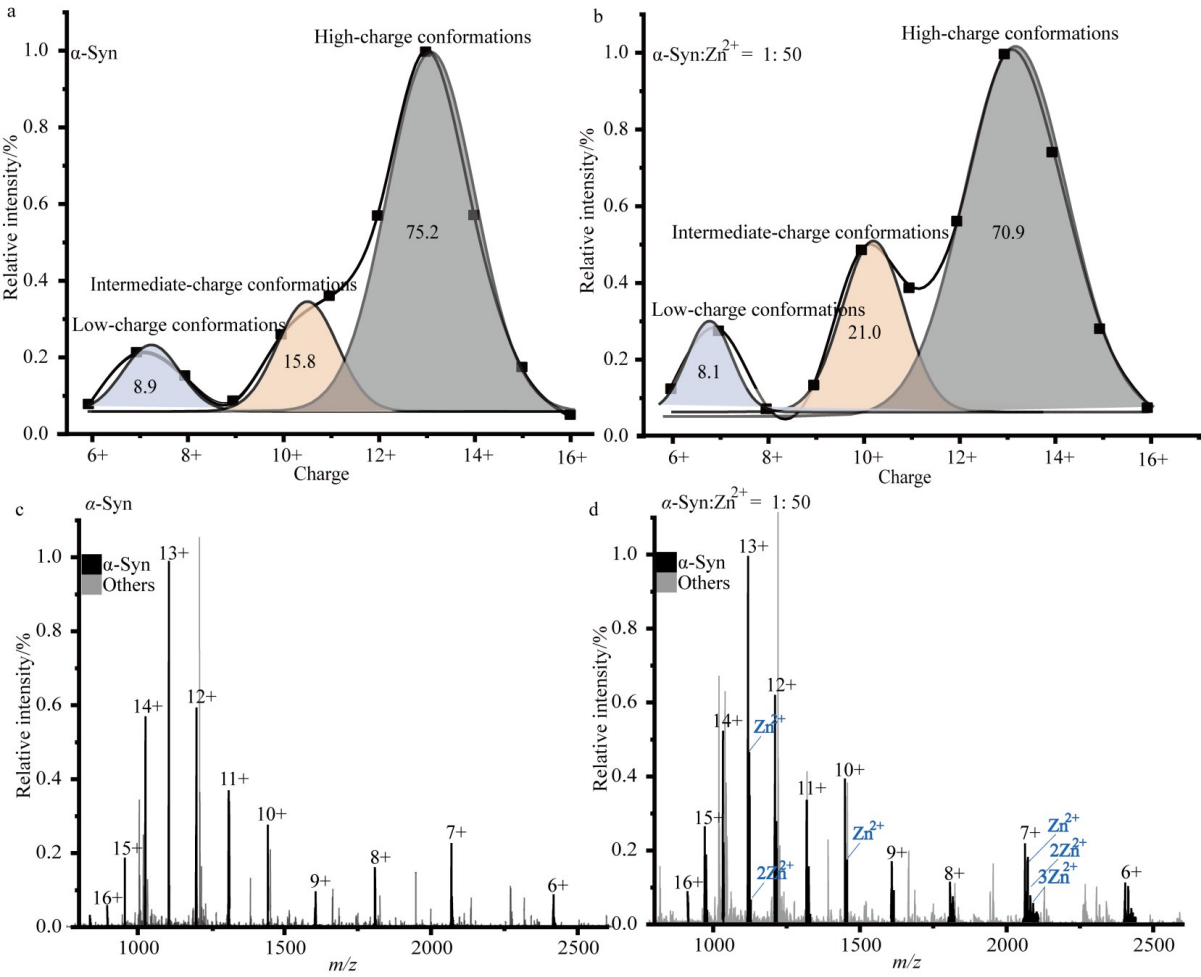
α-Syn结合Zn^2+^（a）前、（b）后的构象分布拟合以及结合Zn^2+^（c）前、（d）后的一级质谱图

**图2:**
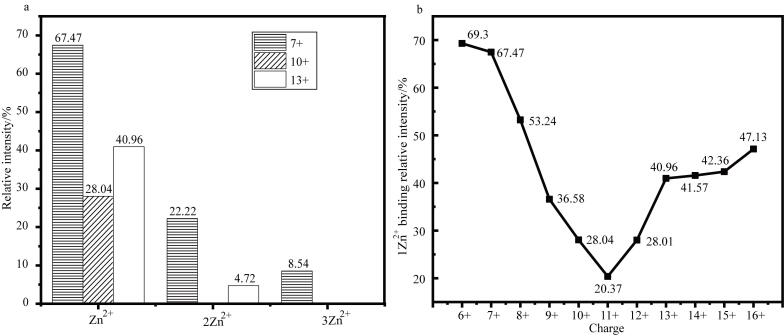
α-Syn结合Zn^2+^：（a）3个典型价态的结合强度分布图；（b）所有价态结合1个Zn^2+^的强度分布图

### 2.2 Zn^2+^对α-Syn的选择性调控

对α-Syn的3个代表电荷态（7+、10+和13+）进行UVPD解离分析，结果表明：在7+、10+和13+电荷态下，分别鉴定得到496、569和494个碎片离子，对应的序列覆盖度依次为85.00%、95.71%和92.86%（图3）。

**图3:**
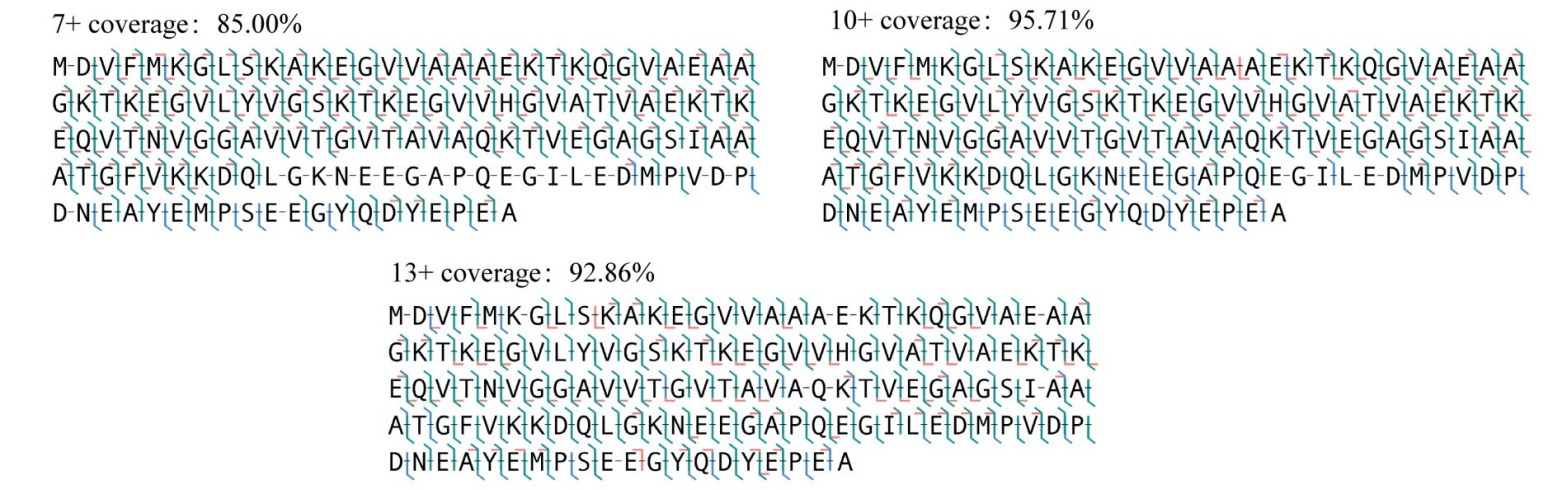
α-Syn不同构象的UVPD覆盖图

为进一步探究Zn^2+^对α-Syn的构象选择性，对Zn^2+^结合前后的代表性价态进行UVPD解离分析。结果显示，Zn^2+^结合后各构象的FYs整体下降（图4a~4c），这一现象证实了α-Syn结构的紧缩效应，与文献报道的离子淌度实验结果高度一致^［12，16］^。然而，不同构象的ΔFYs均值和变化位点存在显著差异（图4a~4c）。此外，按照α-Syn的结构区域划分，对Nterm、NAC和Cterm 3个区域的ΔFYs均值进行计算，发现这3个区域的ΔFYs均值也存在显著差异。

**图4:**
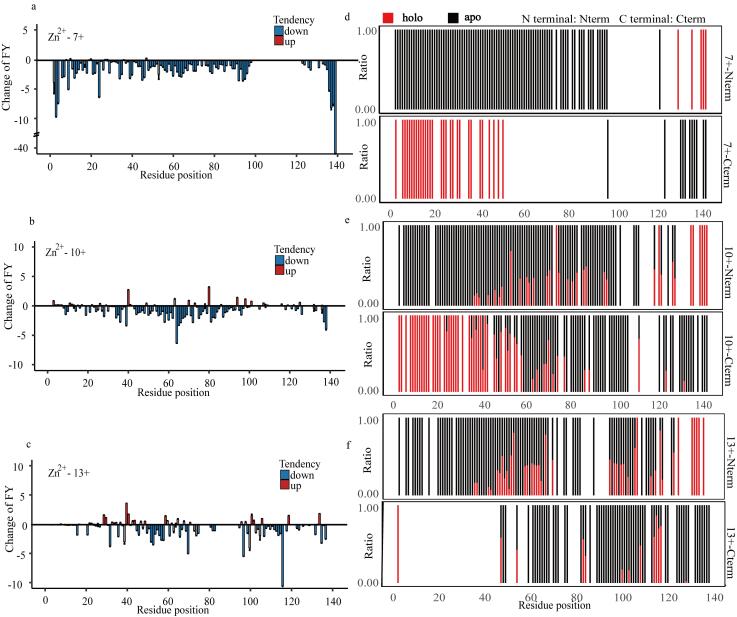
UVPD分析不同构象（7+、10+、13+）的**α-**Syn

低价态构象（7+）与Zn^2+^结合后，FYs下调的主要区域为AA1~100、AA120~140，所有变化位点的ΔFYs均值为-1.69（图4a）。Nterm、NAC区域、Cterm变化位点的ΔFYs均值分别为-1.81、-1.48和-1.48（图4a）。中间价态构象（10+）与Zn^2+^结合后，FYs下调的主要区域为AA5~100、AA120~140，所有变化位点的ΔFYs均值为-0.95（图4b）。Nterm、NAC区域、Cterm变化位点的ΔFYs均值分别为-0.92、-1.41和-0.46（图4b）。高价态构象（13+）与Zn^2+^结合后，FYs下调的主要区域为AA20~80、AA96~140，所有变化位点ΔFYs均值为-0.93（图4c）。Nterm、NAC区域、Cterm变化位点的ΔFYs均值分别为-0.93、-0.98和-0.73（图4c）。

为进一步确认不同构象结合Zn^2+^后的差异，对3种构象的结合Zn^2+^（holo）碎片与未结合Zn^2+^（apo）碎片比值进行分析。结果显示，不同构象的holo碎片分布特征存在显著差异（图4d和4e）：在低价态构象中，Cterm的holo碎片主要分布在AA1~50区域，Nterm主要分布在AA120~140区域，二者分布区域无重叠（图4d）；中间价态构象的Cterm的holo碎片分布区域扩展至AA1~130，Nterm的holo碎片分布于AA40~140，二者主要重叠于AA40~80区域，该区域与已知的Zn^2+^配位结合位点高度吻合（图4e）；高价态构象的Cterm的holo碎片同样分布于AA1~130，Nterm的holo碎片分布于AA40~140，但二者的重叠区域进一步扩大至AA40~130，且碎片分布较为分散（图4f）。

结合一级谱数据与UVPD分析结果进行综合解析表明：低价态构象的Zn^2+^结合数量最多、结合强度最高（图2），结合Zn^2+^后，其Cterm区域ΔFYs下降最为显著（图4a），且Nterm的holo碎片仅分布于AA120~140区间（图4d），其holo碎片总数最少（表1）；中间价态构象仅结合1个Zn^2+^（图2）且结合强度最弱，Zn^2+^结合后，AA40~80区域的ΔFYs下降最为明显（图4b），Nterm和Cterm的holo碎片主要在该区段重叠分布（图4e），但保留了最多的holo碎片（表1）；高价态构象的Zn^2+^结合数量和强度介于低、中价态之间（图2），Zn^2+^结合后，在Cterm和AA40~80区域均呈现显著的ΔFYs下降（图4c），且Nterm和Cterm的holo碎片重叠区域扩展至AA40~130范围（图4f），其holo碎片数量也处于中间水平（表1）。

**表1:** α-Syn结合Zn^2+^后的holo碎片数量

Conformation	Nterm holo	Cterm holo	Sum
Low-charge （7+）	6	31	37
Intermediate-charge （10+）	34	55	89
High-charge （13+）	40	13	53

研究表明，α-Syn中Zn^2+^的主要结合位点包括AA40~80区段（该区域含有可与Zn^2+^形成配位键的组氨酸残基）和CtermAA96~140（强酸性区域，可通过静电相互作用与Zn^2+^形成金属盐）^［10，12，16］^。考虑到配位作用的结合能普遍高于静电作用^［28，29］^，我们推测在UVPD解离下Zn^2+^与Cterm的静电相互作用易被破坏^［30，31］^，导致其较难保留holo碎片，而AA40~80区段的配位作用则更有助于holo碎片的保留。本实验观察到的ΔFYs下降最显著区域、Nterm和Cterm的holo碎片的重叠分布区以及holo碎片数量与文献报道的结合位点高度吻合（图4）。基于此，我们可以推断不同构象的α-Syn与Zn^2+^的结合模式存在差异：低价态构象主要通过Cterm酸性区域的静电相互作用与Zn^2+^结合 ；中间价态构象主要依赖AA40~80区段的配位作用与Zn^2+^结合；而高价态构象与Zn^2+^结合同时涉及静电和配位相互作用两种机制。

## 3 结论

本研究采用nMS联用193 nm UVPD解离技术，实现了对α-Syn构象异质性及其与Zn^2+^结合特征的高分辨率分离与结构解析。该方法充分结合了nMS在保持蛋白天然构象方面的优势与193 nm UVPD在结构域特异性断裂中的高灵敏探测能力，使我们得以从分子层面深入揭示α-Syn与Zn^2+^结合的构象选择性机制。研究结果表明，α-Syn可划分为低、中、高3种构象亚群，且各亚群与Zn^2+^的结合表现出明显的结构依赖性，突显了其构象对金属离子结合的显著影响。本研究展示了nMS-UVPD技术在IDPs与配体相互作用研究中的高分辨能力与机制探测深度。该方法不仅为深入理解Zn^2+^在α-Syn相关神经退行性疾病中的作用提供了重要理论支持，也为IDPs结构功能关系的研究开辟了新的技术路径与分析范式。
